# The Use and Abuse of Gold as a Filling Material

**Published:** 1884-01

**Authors:** A. N. Roussel

**Affiliations:** Newburgh, N. Y.


### THE USE AND ABUSE OF GOLD AS A FILLING MATERIAL.

BY A. N. ROUSSEL, D. D. S., NEWBURGH, N. Y.

READ BEFORE THE SECOND DISTRICT DENTAL SOCIETY.

The first consideration which presents itself to the intelligent judgment of the dentist when about to fill a tooth, is, what is the best material to use for the purpose?

It has been thought for a long time that, in the majority of cases, gold was the only fit substance. Certainly it will make a good filling, and the use of it in the hands of capable operators has done great good; on the contrary, the abuse of it in the *same hands* has done much harm.

There is a general impression prevalent among our patients that gold is *always* the best material with which to fill teeth, simply because *it is gold*, and they would always prefer it were it not for the extra expense. This line of reasoning on their part is solely an association of ideas, and it is not an unfrequent thing for unscrupulous dentists to encourage this impression, for the purpose of obtaining large fees.

As a result of this false reasoning on the part of the patient, and the cupidity of the dentist, we have a real abuse of gold, and that to not a limited extent.

As to the legitimate use of gold: If obliged to establish some definite rule in this respect, or in other words to be asked to draw a line as to where the use of gold should begin, and where it should stop, I would say, fill all the anterior teeth with that material, stopping at the bicuspids. Back of the cuspids use something else, amalgam chiefly. This, it must be understood, is no cast iron rule. Like all laws it is subject to exception and modification, for thousands of bicuspids and molars have been successfully filled with gold.

But in the case of simple crown cavities, in those teeth in which the pulps are alive and healthy, I cannot conceive of one that cannot be as successfully filled with amalgam, as with gold.

The reasons for preferring amalgam to gold in the cases referred to are sufficient. The ability of the amalgam to preserve the tooth as well as gold being admitted, what advantages have the combination of baser metals over the nobler? I answer, three of the most powerful ones we can conceive of. It requires less time, less nervous exhaustion, and less money. All of these considerations particularly interest the patient. It will never do to forget that we owe our patients something besides our mere professional services, for to them is due sympathy, consideration and *economy*.

One thing to which I wish particularly to direct your attention concerning the abuse of gold, is the building of crowns upon the devitalized remains of what was once a tooth. This is an operation that should be strongly denounced.

A tooth that comes to us devitalized, or which we devitalize, is always a doubtful one, for what is the removal of a pulp from a tooth but a matter of guess-work? On several occasions I have been quite disgusted by the arrogance and positiveness with which some operators assert that they always remove *all* the pulp. If, after repeated attempts with the finest broach, no more pulp can be brought away, we lay the flattering unction to our soul that it must be because there is no more to bring away. Most decidedly a matter of inference! How often do we leave enough of this nerve filament behind to engender days of agony for our patients, and no end of trouble for ourselves!

Statistics are not wanted on the subject, and you would not care to hear them.

I do not think there is a gentleman in this room who will positively assert that he *knows* when he has removed all the pulp from an upper or lower molar. Therefore, this being a doubtful operation, and one liable to give both patient and operator annoyance in the future, such a tooth should not have a large and expensive gold plug put in it.

Any devitalized tooth requiring two books of gold to fill it, or rather to build it into the semblance of a tooth, is not fit to fill; it is a case for an artificial substitute, and there is nothing so thoroughly indicated as a good tooth crown, either of gold or porcelain, and of which we have a great variety.

For the anterior teeth, where not more than a third or a quarter of the crown is left, either latitudinally or longitudinally, cut it off and attach a porcelain crown, and you will have a result that you can never hope to attain with a gold plug.

What have we to expect after having built a gold crown on a devitalized tooth ? For the worst an abscess, and all the misery that entails, with probable loss of the tooth ; for the best a slow, low grade of chronic inflammation, giving the patient no peace, while for months he is induced to tolerate the slow torture, in the hope that it will " wear away," a phrase behind which we are too prone to dodge.

There are exceptions to this rule of devitalized teeth being comfortable after having crowns built on them, but they only prove the law. Naturally, the question presents itself, in what way would a porcelain or hollow gold crown be superior to a devitalized tooth ?

It would possess the following advantages :—Probably not more than one-eighth or one-fourth the time would be occupied in attaching the crown to the root or roots, that it would take to build one of gold ; very much less pain and nervous exhaustion to the patient would be the consequence, and decidedly very much less money to pay for it would be demanded. Again, if the root or roots ever gave any after trouble the crown could be easily removed, the tooth subjected anew to treatment, and the crown replaced. Of course this could not be done with a crown that had been built up of gold. You will please observe that the reasons advanced for using the artificial crown on devitalized teeth, are identical with those that I gave for using amalgam back of the bicuspids, with the exception of the last one referring to the matter of treatment.

In connection with this matter of building crowns on devitalized teeth, allow me to say that it has always been a mystery how students of dental pathology could be so inconsistent as to carefully and skillfully treat a case of alveolar abscess for two or three months or longer, and having finally got the root or roots into something like a normal condition, to pound two or more books of gold on those lately convalescing roots, occupying from four to six hours in the performance of the operation, thereby inducing the conditions which they have had so much difficulty in quelling, only to erect a golden monument to their inconsistency and lack of judgment.

